# Thumb adductor muscle thickness used in the nutritional assessment of chronic kidney disease patients under conservative treatment

**DOI:** 10.1590/2175-8239-JBN-2018-0122

**Published:** 2018-09-21

**Authors:** Priscila Moreira de Lima Pereira, Íris Teixeira Soares, Marcus Gomes Bastos, Ana Paula Carlos Cândido

**Affiliations:** 1 Universidade Federal de Juiz de Fora, Instituto de Ciências Biológicas, Departamento de Nutrição, Juiz de Fora, MG, Brasil.; 2 Universidade Federal de Juiz de Fora, Faculdade de Medicina, Departamento de Clínica Médica, Juiz de Fora, MG, Brasil.

**Keywords:** Anthropometry, Nutrition Assessment, Muscles, Renal Insufficiency, Chronic, Conservative Treatment, Antropometria, Avaliação Nutricional, Músculos, Insuficiência Renal Crônica, Tratamento Conservador

## Abstract

**Introduction:**

Evaluate the association between the thumb adductor muscle thickness and the patient's nutritional status, and propose cutoff points for muscle mass depletion in elderly patients with chronic kidney disease (CKD) under conservative treatment. Epidemiological and cross-sectional study, including patients with CKD stages 3 to 5, older than 60 years. Socioeconomic, clinical, physical activity and anthropometric data was obtained. TAMT was described and compared according to CKD stage, socioeconomic data, physical activity, nutritional status and correlated with age, glomerular filtration rate and anthropometric variables. Receiver Operating Characteristic (ROC) curves were produced, considering the lean tissue index classification as reference. The cut-off point was defined by the Youden index.

**Results:**

We evaluated 137 individuals. The TAMT was lower in malnourished and/or depleted muscle mass individuals; among males it was higher among those who practiced physical activities (p <0.05). This measure was moderately correlated with BMI, calf and brachial circumferences, lean body tissue, lean tissue index and body cell mass (r <0.7); negatively with age (r = -0.34). The ROC curve analysis determined cut points of 15.33 mm for females and 20.33 mm for males, with 72.22% and 62.50% accuracy, respectively.

**Conclusion:**

TAMT is used to estimate muscle mass and we suggest the cutoff point is useful to rule out the likelihood of muscle mass depletion. It is recommended that it be used in a complementary way in nutritional assessment.

## Resumo

**Introdução:**

Avaliar a associação da espessura do músculo adutor do polegar (EMAP) com o estado nutricional e propor pontos de corte para depleção de massa muscular em idosos portadores da doença renal crônica (DRC) em tratamento conservador.

**Metodologia:**

Estudo epidemiológico, delineamento transversal, incluindo portadores de DRC estágios 3 a 5, acima de 60 anos. Obteve-se dados socioeconômicos, clínicos, prática de atividade física e antropométricos. A EMAP foi descrita e comparada de acordo com o estágio da DRC, dados socioeconômicos, atividade física e estado nutricional e correlacionada com idade, taxa de filtração glomerular e variáveis antropométricas. Foram produzidas curvas Receiver Operating Characteristic (ROC), considerando como padrão de referência a classificação do índice de tecido magro. O ponto de corte foi definido pelo índice Youden.

**Resultados:**

Avaliou-se 137 indivíduos. A EMAP foi inferior nos desnutridos e/ou com depleção de massa muscular; no sexo masculino foi superior nos praticantes de atividade física (p<0,05). A medida se correlacionou moderadamente com IMC, circunferência da panturrilha e braquial, tecido de massa magra, índice de tecido magro e massa celular corporal (r<0,7), e negativamente com a idade (r=-0,34). A análise da curva ROC determinou pontos de corte de 15,33 mm para o sexo feminino e 20,33 mm para o masculino, com acurácia de 72,22% e 62,50%, respectivamente.

**Conclusão:**

A EMAP serve para estimar a massa muscular, e o ponto de corte sugerido é útil para afastar a probabilidade de depleção de massa muscular. Recomenda-se que seja utilizada de forma complementar na avaliação nutricional.

## Introduction

Chronic kidney disease (CKD) is increasingly prevalent in many countries, becoming one of the major public health challenges with significant economic and social impacts.^1^ An overall prevalence of 11% to 13% is estimated, considering all stages.^2^

Malnutrition in CKD patients is common, and the probability increases linearly with decreased glomerular filtration rate (GFR).^3^ It bears a multifactorial etiology, including factors such as anorexia, metabolic acidosis, increased oxidative stress, inflammatory cytokines, increased catabolism and decreased protein synthesis.^4^^,^ 5 It is one of the main factors that adversely affects prognosis and is associated with longer hospitalization, higher morbidity and higher mortality.^6^ The changes that occur in body composition and functional capacity, such as decreased muscle mass and decreased muscle function and strength,^7^^,^ 8 ^,^ 9 are related to factors such as depression, cardiometabolic complications, poor quality of life and bad prognosis, making the nutritional assessment of these individuals relevant.^10^

Lean mass assessment presents limitations, since changes in body water volume and bone mass in patients with the disease contribute to errors in body composition assessment.^11^ Thus; new anthropometric measures are introduced to fill existing gaps in practicality, cost, reliability and reproducibility, such as the Thumb Adductor Muscle Thickness (TAMT).^12^

TAMT evaluation is a simple, low-cost, and non-invasive procedure. Such a muscle is the only one that can be directly measured. It is influenced by nutritional status, energy catabolism and physical inactivity; It is minimally interfered by body fat and body water^12^^,^ 13 ^,^ 14 ^,^ 15 and has a correlation with lean mass,^13^^,^ 14 ^,^ 16 ^,^ 17 ^,^ 18 being useful to help monitor nutritional status.^12^^,^ 14 ^,^ 19

The present study aims to evaluate the association of TAMT with nutritional status, and to propose cutoff points for the evaluation of muscle mass depletion in elderly patients with CKD under conservative treatment.

## Methods

### Study design and sample selection

A cross-sectional, epidemiological study in which conservative treatment of CKD patients in stages 3 to 5 of both genders, aged 60 and over, was carried out at the State Center for Specialized Care (CEAE) Centro Mineiro de Ensino e Pesquisa em Nefrologia (IMEPEN) in Juiz de Fora, Minas Gerais, Brazil. The site is a referral center and covers a population of 837,991 people, who live in 37 municipalities in the region.^20^

We used the Epi InfoTM software (6.04 version, Centers for Disease Control and Prevention, USA) for sample calculation. We considered the population living in the cities covered by the service,^20^ the prevalence of the disease in stages 3 to 5 (10.6%),^2^ standard error of 2%, confidence level of 99% and 20% of losses, totaling one sample of 120 individuals.

The inclusion criteria were: to have CKD in stages 3 to 5 under follow-up at CEAE/IMEPEN; age 60 years or older and not be the first on-site visit. Exclusion criteria were: presence of hypermetabolic diseases; fracture in one hand; amputation of any limb; be wheelchair user and/or use a pacemaker. Participants who met the inclusion criteria were randomly selected from the attendance schedule. Then, by means of a telephone contact, the procedures were carried out by the active search, sensitization and clarification about the project and the scheduling of the evaluations.

In accordance with ethical issues, approval from the Institutional Ethics Committee (opinion: 1,323,441, CAAE: 48067815.2.0000.5260) was obtained, and the participants signed a Free and Informed Consent Form.

### Study variables

Initially, the participants answered a questionnaire containing socioeconomic information, presence of recent illnesses, injuries and/or fractures in the hands, dominant side and practice of physical activity, being considered physically active those who reported at least 150 minutes of weekly exercise practice.^21^

Their weight was measured on an Tanita Ironman (tm) Scale (model BC-553; Tanita Corporation, Japan). For height measurement we used the Alturexata(r) Estadiometer (Alturexata, Brazil). Body Mass Index (BMI) was calculated and classified according to Lipschitz,^22^ as recommended by the Brazilian Ministry of Health.^23^

Calf circumference (CC) was measured with the individual sitting, with the knee flexed at a 90º angle, and the tape was positioned horizontally in the larger diameter area of the left calf. Values below 31 cm were classified as muscle mass depletion.^24^ Brachial circumference (BC) was measured in the left arm at the midpoint between the acromion and the olecranon. The Frisancho recommendations were considered.^25^ The tricipital skin fold (TCF) was measured in the posterior midline of the left arm, between the acromion and the olecranon, in a triplicate, and we took the simple arithmetic mean between the two closest values. Subsequently, the brachial muscle circumference (BMC) was calculated using the Harrison *et al*. equation,^26^ and the adequacy was determined according to Frisancho.^25^

For body composition assessment we used the Body Composition Monitor(r) (BCM model; Fresenius Medical Care) tetrapolar bioimpedance, which distinguishes muscle mass from pathological fluid overload,^27^ which is in accordance with such methods considered gold standard methods, such as double x-ray emission densitometry (DEXA),^28^ and is specific for patients with kidney failure, and is applicable in all stages of the disease.^29^ We obtained the following data: lean mass tissue (LMT), which represents the body mass without fat tissue and excess extracellular water; lean tissue index, which is calculated by the quotient between LMT/height,² and the body cell mass (BCM), which consists of the metabolically active cell mass, excluding the extracellular fluid of this tissue. The results were classified according to the manufacturer's recommendations.^30^ Prior to testing, the participants were instructed to fast for eight hours, not to exercise, not to consume alcohol and foods containing caffeine in the 12 hours before the test; wear light clothing; and remove metal objects at the time of evaluation.

TAMT was measured with the individual sitting down, with hands relaxed and on the knees and arms resting on the thigh, with the elbow flexed by 90° approximately. Participants were instructed to keep their thumbs apart at an angle of approximately 90° with the index finger. The Lange(r) analog adipometer (Beta Technology Inc.(r), USA) was applied to the adductor muscle of the thumb situated at the apex of the imaginary triangle formed by the extension of the thumb and index finger.^12^ Measurements were performed on both hands in triplicates, and the mean of the closest values was considered.

GFR was calculated from the creatinine test using the CKD-EPI (Chronic Kidney Disease Epidemiology Collaboration)^31^ equation and classified in stages according to KDIGO (2012).^32^

All the data was collected in a single evaluation, individually, by a properly trained team supervised by a senior researcher.

### Statistical analysis

Firstly, exploratory analyzes were performed to check data integrity and coherence. The quantitative variables were evaluated for the presence of outliers and the type of distribution by the Kolmogorov-Smirnov test.

Descriptive analysis of the sample was performed according to gender. Continuous variables with normal distribution were represented by mean ± standard deviation and compared using the Student's t-test; the non-parametric variables were described with median, minimum and maximum values ​​and the Mann-Whitney test was performed. The categorical variables were presented with absolute and relative frequencies and the Pearson's chi-square test was used.

TAMT mean values were described and compared by the Student's t-test according to CKD stage, socioeconomic data, physical activity practice and nutritional status. Correlations between TAMT with age, GFR and anthropometric variables were assessed by the Pearson or Spearman correlation tests. Weak correlations were those less than 0.30; moderate, between 0,30 and 0,70; and strong, when higher than 0,70. The analyses were performed using the Statistical Package for the Social Sciences (version 17.0; SPSS Inc., Chicago, IL, USA), with a significance level set at 5.0%.

Receiver Operating Characteristic (ROC) curves were produced using the classification of the lean tissue index less than or equal to the 10th percentile as a reference, according to gender and age.^30^ The cutoff point was defined using Youden's index, which represents the value of a better balance between sensitivity and specificity. The values of accuracy, sensitivity, specificity, positive and negative predictive values and their respective 95% confidence intervals (95% CI) were evaluated using the MedCalc(tm) software (version 17.9.7).

## Results

The sample consisted of 137 individuals, 60.6% male, with a mean age of 72.89 ± 7.66 years, with a median monthly income of approximately one minimum wage ($ 937.00 Reals or $ 293.73 US Dollars ) and low schooling - the majority (67.6%) had incomplete elementary education and only 1.5% had completed higher education.

Regarding CKD stage, 14.7% were classified in stage 3A; the majority (52.2%) in stage 3B; 27.2%, at 4; and only 5.9% in stage 5. In addition, a large part of the sample had other CKD-associated morbidities, such as hypertension (86.9%), DM (51.1%) or both (48.2%). Regarding nutritional status, there is a high number of overweight individuals (60.3%) and a low prevalence of malnutrition (9.6%), according to BMI.

Table 1 depicts the characteristics of the sample according to gender. It is noteworthy that women had higher BMI values and lower muscle mass (evaluated by CC, BMC, bioimpedance and TAMT) in relation to men.

**Table 1:** General characteristics of patients carriers of chronic renal disease in conservative treatment

Variable	Female	Male	p
Age (years)	74,17 ± 7,22	72,30 ± 8,12	0,171 *
Monthly income (real)	937,00 (150,00 – 2000,00)	1000,00 (937,00 – 5000,00)	0,005 †
Monthly income (dollar)	293,73 (47,02 – 626,96)	313,48 (293,73 – 1567,40)	0,005 †
Schooling (incomplete elementary school)	43 (79,6)	61 (73,5)	0,542 §
Physical activity practice	19 (35,2)	32 (38,1)	0,857 §
Presence of DM	27 (50)	43 (51,2)	1,00 §
Presence of SAH	50 (92,6)	70 (83,3)	0,128 §
Presença de SAH e DM	27 (50)	39 (46,4)	0,729 §
GFR (ml/min/1,73m²)	33,29 ± 11,02	34,36 ± 11,73	0,596 *
BMI (kg/m²)	29,85 ± 5,17	27,79 ± 4,89	0,019 *
CC (cm)	35,18 ± 3,37	36,85 ± 3,83	0,011 *
BC (cm)	32,00 (21,00 – 38,00)	30,00 (23,50 – 39,00)	0,065 †
BMC (cm)	23,29 ± 2,42	24,77 ± 3,18	0,004 *
LTM (kg)	29,86 ± 8,37	42,65 ±9,03	<0,001*
Lean tissue index (kg/m²)	12,70 (8,00- 24,90)	15,62 ± 3,06	<0,001†
BCM (kg)	16,10 (7,90 – 33,40)	24,58 ± 6,27	<0,001 †
APMT dominant (mm)	17,22 ± 3,65	20,45 ± 5,10	<0,001 *
APMT not dominant (mm)	16,44 ± 3,92	19,74 ±4,90	<0,001 *

* Test T Student.

† Test Mann – Whitney.

§ Test Chi square.

Continuous variables with normal distribution, represented by mean values ± standard deviation. Non-parametric variables, represented by median, minimum and maximum values. Categorical variables, represented according to the absolute and relative frequencies.

DM: diabetes mellitus, SAH: systemic arterial hypertension, GFR: glomerular filtration rate, BMI: body mass index, CC: calf circumference, BC: brachial circumference, BMC: brachial muscle circumference, LTM: lean tissue mass; BCM: body cell mass, APMT: adductor pollicis muscle thickness.

After analyzing TAMT in both hands, it was found that they were similar and strongly correlated in females and males (r = 0.88 and r = 0.84, respectively). Therefore, for the subsequent analyses, we evaluated only dominant hand's TAMT.

Table 2 depicts TAMT's mean values and standard deviation according to CKD stage, socioeconomic data, physical activity practice and nutritional status. The measure was lower in individuals of both genders classified as underweight according to BMI, and with muscle mass depletion according to CC, BMC and lean tissue index. TAMT was higher among the active individuals.

**Table 2:** Adductor pollicis muscle thickness according to the stage of chronic kidney disease, socioeconomic data, physical activity practice and nutritional status

	**APMT**					
	**Female**			**Male**		
	n	Mean ± SD	*p* *	n	Mean ± SD	*p* *
Stage of CKD						
Stage 3 (A or B)	37	17,59 ± 4,03	0,201	54	20,35 ± 5,57	0,776
Stage 4 or 5	17	16,41 ± 2,59		28	20,69 ± 4,35	
Schooling						
Incomplete elementary school	43	17,08 ± 3,15	0,681	61	20,30 ± 5,41	0,550
Complete elementary school	11	17,79 ± 5,36		21	21,09 ± 4,34	
Monthly income						
≤ 937,00 real or 293,73 dollars	40	17,55 ± 3,50	0,228	41	19,77 ± 5,05	0,228
> 937,00 real or 293,73 dollars	14	16,29 ± 4,03		42	21,13 ± 1,17	
Physical activity practice						
Active (≥ 150 minutes / week)	44	17,10 ± 3,68	0,605	59	21,19 ± 5,34	0,042
Sedentary (< 150 minutes / week)	10	17,77 ± 3,68		24	18,67 ± 4,13	
BMI						
≥ 22kg/m²	51	17,55 ± 3,44	0,006	72	21,15 ± 4,71	0,001
< 22kg/m²	3	11,67 ± 3,06		10	15,50 ± 5,81	
CC						
≥ 31cm	49	17,61 ± 3,54	0,013	78	20,91 ± 4,81	0,001
< 31cm	5	13,40 ± 2,57		5	13,37 ± 5,17	
BC						
≥ 90% adequacy	51	17,55 ± 3,44	0,006	69	21,25 ± 4,72	0,001
< 90% adequacy	3	11,67 ± 3,06		14	16,53 ± 5,40	
BMC						
≥ 90% adequacy	48	17,14 ± 3,36	0,640	45	20,84 ± 4,73	0,458
< 90% adequacy	5	17,89 ± 5,89		38	20,00 ± 5,59	
Lean tissue index						
≥ p10	35	18,22 ± 3,43	0,005	56	21,48 ± 5,05	0,043
< p10	19	15,39 ± 3,40		24	19,47 ± 3,44	

* Teste t de Student.

SD: standard deviation, APMT: adductor pollicis muscle thickness, CKD: chronic kidney disease, BMI: body mass index, CC: calf circumference, BC: brachial circumference, BMC: brachial muscle circumference.

The female TAMT was positively and moderately correlated to BMI, CCC, BC, lean mass and BMC, and weakly to LMT and lean tissue index. Among males, there were positive and moderate correlations with BMI, CC, BC, lean mass, LMT and BMC, weak correlation with lean tissue index, and a negative and moderate with age (Table 3).

**Table 3:** Correlation between the adductor pollicis muscle thickness of the dominant hand with age, glomerular filtration rate and anthropometric variables

	APMT					
	Female		Male		Total	
	R	P	R	P	R	P
Age (years)	-0,072	0,605 *	-0,415	<0,001 *	-0,335	<0,001 *
GFR (ml/min/1,73m²)	0,157	0,256 *	0,026	0,818 *	0,074	0,039 *
BMI (kg/m²)	0,492	<0,001 *	0,524	<0,001 *	0,400	<0,001 *
CC (cm)	0,568	<0,001 *	0,496	<0,001 *	0,544	<0,001 *
BC (cm)	0,319	0,019 †	0,439	<0,001 †	0,315	<0,001 †
BMC (cm)	0,134	0,335 *	0,102	0,360 *	0,118	0,171 *
LTM (kg)	0,285	0,037 *	0,351	0,001 *	0,489	<0,001 *
Lean tissue index (kg/m²)	0,281	0,040 †	0,267	0,016 *	0,340	<0,001 *
BCM (kg)	0,310	0,023 †	0,319	0,004 *	0,442	<0,001 *

* Pearson correlation;

† Spearman correlation..

APMT: adductor pollicis muscle thickness, GFR: glomerular filtration rate, BMI: body mass index, CC: calf circumference, BC: brachial circumference, BMC: brachial muscle circumference, LTM: lean tissue mass; BCM: body cell mass.

**Figure 1:**
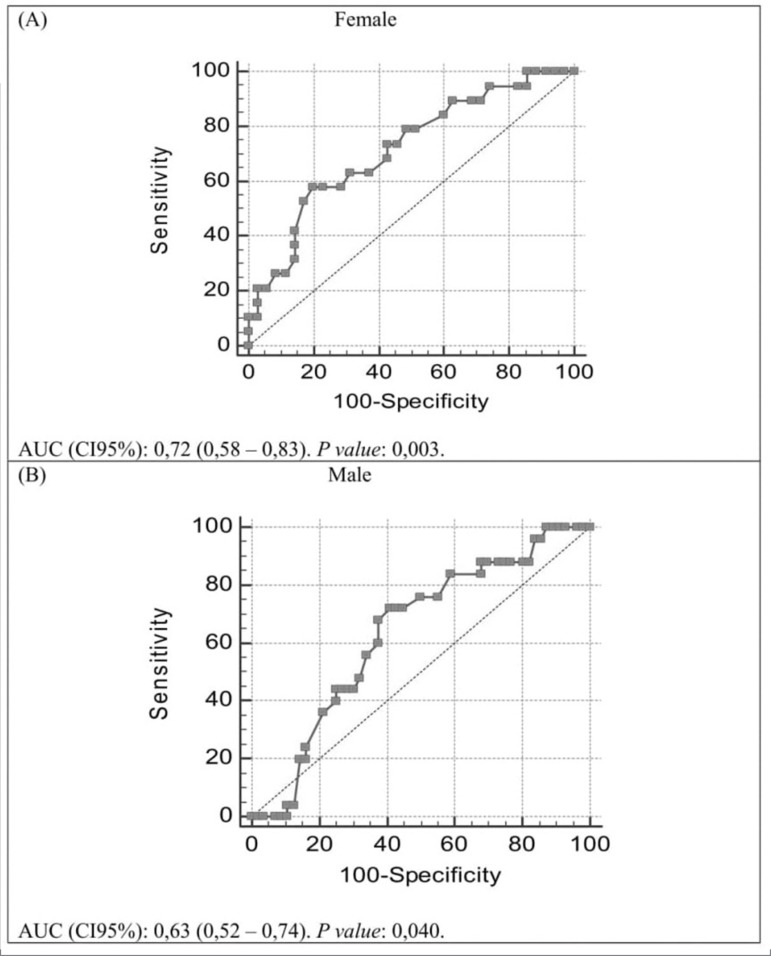
Receiver Operating Characteristic (ROC) curve to discriminate muscle mass depletion according to the thumb adductor muscle thickness.

The ROC curve analysis determined cutoff point of 15.33 mm for women and 20.33 mm for males. Being more specific for women (80.0%) and more sensitive for men (70.8%). It should be noted that these values had an accuracy of 72.22% and 62.50%, respectively (Figure 1 and Table 4).

**Table 4:** Discriminatory power of the adductor pollicis muscle thickness for depletion of muscle mass in patients with chronic kidney disease

		Female		Male
Cutting point (mm)		15,33		20,33
Youden index		0,38		0,31
Acuracy (%) (95% CI)		72,22 (58,36 – 83,54)		62,50 (50,96 – 73,08)
Sensitivity (95% CI)		57,89 (33,50 - 79,75)		70,83 (48,91 – 87,38)
Specificity (95% CI)		80,00 (63,06 - 91,56).		58,93 (44,98 – 71,90)
PPV (95% CI)		61,11 (42,22 – 77,16)		42,50 (33,01 – 52,58)
NPV (95% CI)		77,78 (66,82 – 85,88)		82,50 (70,89 – 90,13)

CI: confidence interval, PPV: positive predictive value, NPV: negative predictive value.

Considering these cut-off points, 33.3% of the women and 43.4% of the men had muscle mass depletion. Comparing the prevalence of muscle mass depletion, determined by TAMT, with those obtained by other anthropometric measures, we noticed that in females, it was higher than that obtained by CC (9.3%), BC (5, 6%) and BMC (11.1%); however, it was similar to the diagnosis made by the thin tissue index of 35.2%. In males, it was higher than CC (6%), BC (16.9%) and lean tissue index (30%), similar to BMC results (45.8%).

## Discussion

In the present study, TAMT was significantly lower in the individuals classified as malnourished and/or with muscle mass depletion according to several parameters. It correlated with other anthropometric measures, such as BMI, CC, BC, lean mass, BMC, LMT and lean tissue index. The proposed cutoff point had an accuracy of 72.22% for females and 62.50% for males.

The mean TAMT values obtained (20.45 ± 5.10 mm in males and 17.22 ± 3.65 mm in females) were lower than those found in studies with healthy and younger populations, such as the study ran by Gonzalez *et al*.^19^ (whose values were 26.1 ± 4.4 mm and 19.8 ± 3.3 mm for men and women, respectively), and Bielemann *et al*.^18^ (24.2 ± 4.2 mm for both genders). However, it was higher than those from other studies with healthy subjects, performed by Ghorabi *et al*. (2014)^33^ (the mean values for males and females were 14.6 ± 3.2 mm and 11.2 ± 2.4 mm, respectively) and by Lameu *et al*. (2004)^12^ (men: 12.5 ± 2.9 mm and women: 10.5 ± 2.3 mm). Our findings were also higher than those from other studies with ill individuals, such as outpatient HIV-infected patients,^34^ large-scale gastrointestinal-surgical patients,^14^ inpatients in intensive care units.^35^

No other study evaluating TAMT in patients with CKD on conservative treatment was found in the literature, there were only studies involving patients undergoing dialysis treatment, such as those carried out by Oliveira *et al*.^36^ and Pereira *et al*.^37^, who presented lower mean values than those in this study: 10.0 ± 4.5 mm and 11.9 ± 1.6 mm, for both genders, respectively. This is justified by the fact that at the non-dialysis stage, the prevalence of malnutrition is lower than in the dialysis phase.^38^ In these studies, the authors concluded that TAMT is a promising marker of nutritional status,^37^ and it may be a useful parameter for the early diagnosis of malnutrition, risk assessment for hospitalization and mortality.^36^

Regarding the different TAMT values found in the studies, some considerations should be made. First, sample characteristics such as gender, age, race/skin color, body size, nutritional and health conditions interfere with the results.^12^^,^ 13 ^,^ 14 In this study, although the sample is made of elderly individuals and patients with a disease that depletes muscle mass, the prevalence of overweight individuals was high and malnutrition was low, which may have overestimated the values. Another factor that may cause divergences are the methodological inadequacies, since research identifying very discrepant values may be based on errors related to the calibration and the type of instrument adopted for the assessment,^19^ intra- and inter-examiner variability and the incorrect clamping of the anatomical site, because very low measures represent the thickness of the skinfold near the muscle, and not of the TAMT.^18^

TAMT measurements were different between the genders, being higher in males, as per reported by other authors.^12^^,^ 18 ^,^ 33 ^,^ 34 ^,^ 36 Skeletal muscle mass is influenced by testosterone levels, so men often present a higher muscle density. This measure was also influenced by age, since it was negatively correlated with it (r = -0.335), as well as that identified by Pereira *et al*.,^37^ when evaluating patients with CKD under dialysis (r = -0, 32). It is known that the thickness of this muscle tends to decrease with age, being more significant as of the age of 65 years.^12^^,^ 33 Aging reduces the amount of type-2 fibers because of the neurogenic changes that induce denervation, something that, together with the lower production of mitochondrial adenosine triphosphate, causes a reduction in muscle mass.^39^ Such a fact should be considered in our sample, since the mean age is 72.89 ± 7.66 years.

Another factor that seems to interfere with TAMT is laterality. Although there is no consensus on the side to be evaluated, a large part of the studies opt for the dominant hand, as well as the pioneering work of Lameu *et al*.^12^ One possible justification for this choice would be the fact that the muscle was responsible for the opposition of the thumb, a movement performed in almost all routine activities, being more required in the dominant hand. Thus, there is a tendency to be measured in this hand, since the most exercised muscle tends to atrophy more rapidly in a situation of malnutrition.^13^

The results show evidence that the TAMT can be used as an indicator of muscle mass, since it has remained associated and correlated with other anthropometric measures that evaluate the same compartment (CC, BC, lean mass, LMT, BMC and lean tissue index), similar to that found by other authors.^12^^,^ 33 ^,^ 34 ^,^ 36 However, this should be interpreted with caution, because anthropometric measurements have limitations, such as the tendency to overestimate muscle mass, when compared to a reference standard.^40^

The suggested cutoff point is useful to rule out the probability of muscle mass depletion, since men with TAMT greater than 19.33 mm and women with TAMT greater than 15.33 mm have 82.5% and 77.8%, respectively, likelihood of muscle mass depletion (negative predictive values). However, to confirm the diagnosis of muscle mass depletion, we recommend that the measurement be associated with other anthropometric indicators.

It should be noted that sensitive tests are important for the early detection of muscle mass depletion in CKD patients, and are essential for reducing the risks inherent to the presence of sarcopenia, preventing the fragility syndrome, providing well-being^8^ and achieving a better prognosis.^35^ In addition, there is evidence that a poorer nutritional status at the beginning of dialysis contributes to lower survival and greater likelihood of developing complications.^6^ However, since there is no ideal protocol for the nutritional assessment of these individuals, it is advisable to employ a combination of indicators to improve the nutritional diagnosis accuracy. In general, dual X-ray absorptiometry (DEXA) is suggested as the ideal method to assess muscle mass, but its low availability and high cost limit its use in clinical practice. In this way, alternative methods such as the TAMT gains relevance.

Among the limitations of this study are its cross-sectional nature, which does not enable us to establish the cause and effect relationship and the applicability of the measure to evaluate the clinical prognosis and changes in body composition in the long term; the absence of a gold standard for assessing muscle mass, limiting the validity of the proposed cutoff points, and the fact that the study was designed to evaluate elderly patients with CKD under conservative treatment, limiting the extrapolation of results to other groups. However, although it presents limitations, the study is relevant due to the relevance of the theme and its originality.

## Conclusion

We concluded that TAMT measurement can be used for the nutritional assessment of patients with CKD submitted to conservative treatment, since it has been associated with other anthropometric measures (CC, BC, lean mass, LMT, BMC and lean tissue index), demonstrating that it is effective in estimating muscle mass. The adoption of the proposed cutoff point is useful to rule out the probability of muscle mass depletion and should be used in a complementary way in nutritional assessment.
